# Personal vehicle use and food security among US adults who are primary shoppers for households with children

**DOI:** 10.1007/s44187-023-00048-6

**Published:** 2023-06-12

**Authors:** Curtis Jalen Antrum, Molly E. Waring, Kristen Cooksey Stowers

**Affiliations:** 1 University of Connecticut, Department of Allied Health Sciences, 358 Mansfield Rd, Storrs, CT 06269 USA; 2 UConn Rudd Center for Food Policy and Health, Hartford, CT USA

**Keywords:** Food Security, Vehicle access, Food access, Social determinants, Grocery shopping

## Abstract

In 2020, 2.9 million households with children were food secure. Previous studies have demonstrated that reduced car access may contribute to issues of food security. This study examines whether using a personal vehicle by primary shoppers of households with children is associated with food security. Data were from US adults who were primary shoppers for households with children (N = 997) in the National Health and Nutrition Examination Survey 2017–2018. Participants reported their means of transportation to complete grocery shopping, which was categorized as using a personal vehicle or another mode of transportation. Household food security was measured using the US Food Security Survey Module and categorized as full food security, marginal food security, low food security, or very low food security. Multinomial logistic regression models estimated the association between transportation mode and food security. Adjusted models included age, race/ethnicity, education, and poverty-to-income ratio variables. 3.8% (SE: 0.6%) of US adults who are primary shoppers for households with children did not use a personal vehicle for grocery shopping. US adults who used a personal vehicle for grocery shopping were less likely to report very low food security [22.1% (SE: 7.4%) vs. 8.5% (SE: 1.3%), aOR = 0.4, 95% CI 0.1–1.0]. There were no differences in marginal food security [18.3% (SE: 3.3%) vs. 13.1% (SE: 1.7%), aOR = 0.9, 95% CI 0.6–1.5] or low food security [30.6% (SE: 8.7%) vs. 16.1% (SE: 1.7%), aOR = 0.6, 95% CI 0.2–1.7]. Future research and interventions should address how transportation access may contribute to food security in families with children.

## Introduction

In 2020, 13.8 million households were food insecure, which includes 2.9 million households with children [1]. For households with children, food insecurity is defined as households where parents or guardians are sometimes unable to provide adequate, nutritious food for their children [1]. Food insecure households report lower quality diets with less consumption of fruits and vegetables [2]. Specifically, food insecure adults experienced lower intake of essential micronutrients, such as vitamins A and B-6, zinc, and magnesium [3]. In adults, food security has shown an association with obesity [4] and diabetes [5]. For children, food insecurity is also associated with several adverse health outcomes [6], including asthma [7], anemia [8], and developmental risk [9].

Previous research has examined the associations between food security, perceived food access, and geographic proximity to the nearest grocery store and food security among low-income families in several diverse settings [10–13]. Studies employing geospatial analyses have displayed this relationship, showing that specifically amongst low-income participants, residents with further driving or travel distances had an increased likelihood of experiencing food insecurity [12, 14]. Among food insecure households, perceived food access was lower even when a grocery store was within the defined geographic area [15]. Perceived inability to walk to a grocery store has been associated with lower fruit and vegetable consumption, even when a grocery store was within 1 km [16]. A potential reason for this may be that walking even 1 km presents challenges for those buying groceries for larger households or shoppers who must bring small children on errands.

Reduced car access has been highlighted as one of many reasons why lower income individuals may experience food insecurity [17, 18]. A study from Australia highlighted the importance of access to transportation as a critical factor in determining whether households have sufficient access to food [17]. A study that conducted focus groups with women from West Virginia described caregivers' struggles in accessing consistent transportation, including walking long distances to the grocery store or rushing to catch public transportation [10]. To date, vehicle ownership has been discussed as a key factor contributing to weekly food expenditures and food distress. Yet, strong evidence has not been found linking vehicle ownership specifically to food security [19]. To better inform research and policy related to food security, research examining transportation access among shoppers for families across the United States is needed. This study aims to investigate whether US adults responsible for grocery shopping in households with children who do not use a personal vehicle for grocery shopping are more likely to experience food insecurity than primary shoppers who use a personal vehicle.

## Methods

Data from the National Health and Nutrition Examination Survey (NHANES) 2017–2018 was used for this analysis. NHANES collects nationally representative health data on the United States population [20]. The National Center for Health Statistics approved the NHANES study protocol, where participants provided informed consent to in-home questionnaires and physical examinations. The current analysis did not require IRB approval as it does not meet the criteria for human subjects research.

The analytic sample was comprised of respondents aged 20 + years old who reported that they were the primary shopper for households with children (0–17 years). Participants were asked if they were the person who did the most shopping for food in their family. Response options were “yes” or “no,” and NHANES interviewers were instructed to code respondents who answered “sometimes” or “50/50” as “yes” [21].

Participants were asked how they usually get to the store (or stores) where they do most of their grocery shopping and asked to choose from 8 options. Participants who indicated more than one mode of transportation were instructed to choose their usual or most common method of transportation. Responses were categorized as using a personal vehicle (their own vehicle or a vehicle owned by someone they live with) versus another mode of transportation (a car that belongs to someone outside their household, public transit, taxis, walking, riding a bicycle, or having groceries delivered).

Household food security was measured using the 18-item US Food Security Survey Module developed by the United States Department of Agriculture (USDA) [22]. This module asks a series of questions related to a household's accessibility to healthy food and ability to feed each individual. Based on responses, households are assigned a score between 0 and 18. NHANES established a four-level categorization of food security for households with children: full security (score of 0), marginal food security (1–2), low food security (3–7), and very low food security (8–18).

Participants reported their age, race/ethnicity, and educational attainment. Participant age was categorized into four categories: 18–34 years, 35–49 years, 50–64 years, and 65 + years. NHANES uses a 6-level categorization of race/ethnicity as Mexican American, Other Hispanic, Non-Hispanic White, Non-Hispanic Black, Non-Hispanic Asian, and Other race/multiracial. Educational attainment was categorized into four levels less than high school, high school diploma, some college or associate's degree, or college graduate or higher education.

The Poverty Income Ratio (PIR) is also calculated for each family unit in NHANES.

### Statistical analysis

Statistical analysis was executed using SAS version 9.4 software (SAS Institute Inc., Cary, NC, USA). Sampling weights were used in all analyses, and results are representative of US adults who are the primary shopper for households with children nationally. We described the characteristics of primary shoppers who either did or did not use a personal vehicle in terms of age, race/ethnicity, and educational attainment. Multinomial logistic regression was used to measure the association between personal vehicle use and levels of household food security. In multinomial logistic regression, the outcome is categorical with 3 + categories. Models are simultaneously fit by using maximum likelihood to estimate odds ratios (ORs) and 95% confidence intervals (95% CIs) for each level compared to a common reference level (here, full food security) [23]. Adjusted multinomial logistic regressions included age, race/ethnicity, educational attainment, and poverty-to-income ratio. Finally, we examined the association between transportation and food security using an adjusted multinomial logistic regression among the subpopulation of households below 130% of the federal poverty line.

## Results

Among the 2118 adults aged ≥ 20 years living in a home with children in NHANES 2017–2018, 848 reported that they were not the primary shopper. Of the 1270 US adults who were the primary shopper in a household with children, we excluded those who were missing data on educational attainment (n = 2), mode of transportation used for grocery shopping (n = 75), family poverty-to-income ratio (n = 266), and/or household food security (n = 75), resulting in an analytic sample of 997 adults, representing about 43.6 million US adults.

Overall, 96.2% (SE: 0.6) of US adults who were the primary shopper in households with children used a personal vehicle for grocery shopping (Table 1). The 35–49 age group accounted for 51.2% (SE: 3.4) of those who used a personal vehicle for grocery shopping and 37.3% (SE: 6.1) of primary shoppers who used another mode of transportation. Among primary shoppers who did not use a personal vehicle, 56.2% (SE: 2.9) were Non-Hispanic White, while 11.5% (SE: 5.0) of those who did not use a personal vehicle were Non-Hispanic White. In terms of education, 31.2% (SE: 3.5) of those who use their personal vehicle had completed college or above, compared to 7.5% (SE: 3.6) of those who did not use a personal vehicle having completed college or above.

**Table 1:** Characteristics of US adults who are the primary shopper for households with children in relation to usual transportation used for grocery shopping

	Use personal vehicle for grocery shopping, weighted % (SE)	Do not use personal vehicle for grocery shopping, weighted % (SE)
Sample N	937	60
Weighted N	41.9 million	1.6 million
Age		
20–34 years	31.1 (2.7)	45.8 (5.8)
35–49 years	51.2 (3.4)	37.3 (6.1)
50–64 years	15.0 (2.1)	12.5 (3.9)
65 + years	2.7 (0.6)	4.3 (2.9)
Race/Ethnicity		
Hispanic	19.7 (2.3)	32.4 (7.5)
Non-Hispanic White	56.2 (2.9)	11.5 (5.0)
Non-Hispanic Black	13.3 (2.2)	41.7 (5.9)
Other race/multiracial	10.5 (1.8)	14.4 (5.5)
Education Level		
Less than high school	10.2 (1.4)	29.7 (8.9)
High school diploma or GED	26.0 (2.5)	34.1 (6.2)
Some college or Associate's degree	32.6 (2.7)	30.5 (7.3)
College graduate or above	31.2 (3.5)	7.5 (3.6)

Only 28.9% (SE: 4.7) of US primary shoppers who did not use a personal vehicle had full food security compared to 62.4% (SE: 2.8) of shoppers who used a personal vehicle (Table 2). US adults who are primary shoppers in households with children who used a personal vehicle were less likely to experience very low food security than those who used another means of transportation [22.2% (SE: 7.4%) vs. 8.5% (SE: 1.3%), aOR = 0.4, 95% CI 0.1–1.0]. There were no differences in low [30.6% (SE: 8.7%) vs. 16.1% (SE: 1.7%), aOR = 0.6, 95% CI 0.2–1.7] or marginal food security [18.3% (SE: 3.3%) vs. 13.1% (SE: 1.7%), aOR = 0.9, 95% CI 0.6–1.5; Table 2] between family shoppers in the US who used or did not use a personal vehicle after adjusting for age, race/ethnicity, educational attainment, and poverty-to-income ratio.

**Table 2:** Associations between mode of transportation by primary shopper and food security among US adults who are the primary grocery shoppers for households with children

	Full food security	Marginal food security	Low food security	Very low food security
Full sample (N = 997)				
Use personal vehicle, weighted % (SE) Do not use personal vehicle, weighted % (SE) Crude OR (95% CI) Adjusted^a^ OR (95% CI) Adjusted^b^ OR (95% CI)	62.4 (2.8) 28.9 (4.7) (Reference) (Reference) (Reference)	13.1 (1.7) 18.3 (3.3) **0.3 (0.2–0.6)** 0.7 (0.4–1.3) 0.9 (0.6–1.5)	16.1 (1.7) 30.6 (8.7) **0.2 (0.1–0.5)** 0.5 (0.2–1.2) 0.6 (0.3–1.7)	8.5 (1.3) 22.2 (7.4) **0.2 (0.1–0.5)** **0.2 (0.1–0.6)** **0.4 (0.1–1.0)**
< 130% federal poverty line (N = 451)				
Use personal vehicle, weighted % (SE) Do not use personal vehicle, weighted % (SE) Crude OR (95% CI) Adjusted* OR (95% CI)	35.9 (3.4) 23.1 (5.6) (Reference) (Reference)	18.9 (3.3) 18.7 (4.3) 0.7 (0.3–1.3) 1.0 (0.5–2.0)	27.3 (2.9) 33.3 (11.4) 0.5 (0.2–1.5) 0.6 (0.2–1.6)	17.9 (2.3) 24.9 (9.1) 0.5 (0.2–1.3) **0.3 (0.1–0.7)**

^a^Adjusted for age, race/ethnicity, and educational attainment

^b^Adjusted for age, race/ethnicity, educational attainment, and poverty-to-income ratio

Values in bold signify statisically significant odd ratios where p<0.05

**Fig. 1:**
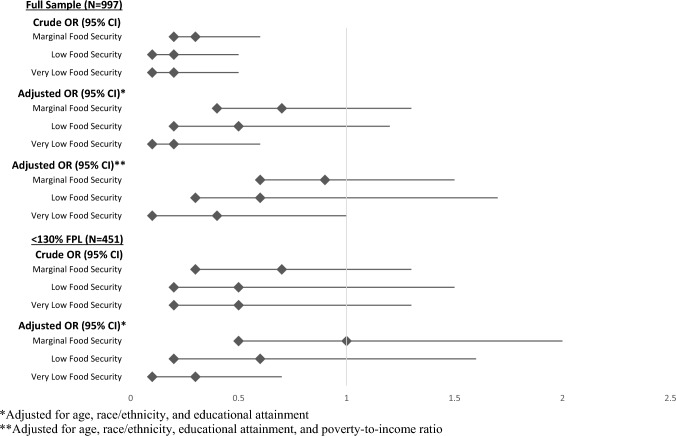
Odds Ratios: Mode of transportation and food security among US adults who are the primary shoppers for households with children. *Adjusted for age, race/ethnicity, and educational attainment. **Adjusted for age, race/ethnicity, educational attainment, and poverty-to-income ratio

Among US adults from households below 130% of the poverty line (N = 451), 8.6% (SE: 1.5%) used transportation other than a private vehicle for grocery shopping. Among these low-income families, 35.9% (SE: 3.4) and 23.1% (SE: 5.6) of family shoppers who used a personal vehicle or other means of transportation, respectively, reported full food security (Table 2). There were no differences in the likelihood of marginal food security [18.9% (SE: 3.3%) vs. 18.7% (SE: 4.3%), OR = 1.0, 95% CI 0.5–2.0] or low food security [27.3% (SE: 2.9%) vs. 33.3% (SE: 11.4%), OR = 0.6, 95% CI 0.2–1.6; Table 2, Fig. 1]. Family shoppers who used a personal vehicle were less likely to experience very low food security than family shoppers who used another means of transportation [17.9% (SE: 2.3%) vs. 24.9% (SE: 9.1%), OR = 0.3, 95% CI 0.1–0.7; Table 2, Fig. 1].

## Discussion

This study examined the relationship between the usual mode of transportation for grocery shopping and household food security in US households with children. We found that family shoppers who did not use a personal vehicle were more likely to report very low food security but not marginal or low food security compared to those who used another means of transportation. These results add to previous evidence that reduced vehicle access is one of many factors associated with food insecurity for households with children in the United States. In recent years, many issues related to the social determinants of health were exacerbated by the Coronavirus pandemic. To be better prepared in the event of another societal or economic downturn, researchers ought to understand the mechanisms through which these disparities occur. The results of this paper help clarify the key role of vehicle ownership in food access.

Geographic proximity to a grocery store may not be the only factor contributing to food access issues for food insecure households [15, 18]. This study adds to the literature examining food security and access by highlighting the link between transportation mode and family food insecurity, regardless of proximity to food outlets. Previous studies have shown that increased physical constraints are a challenge for many low-income individuals experiencing food security [17]. A deeper understanding of how vehicle access relates to family food security may help researchers help shape policy to increase food access to those who need it. The current study's findings align with previous studies' findings, which emphasize difficulties in food access for those who do not have access to a vehicle in car-dependent communities [10, 24, 25].

Issues of food access in specific food environments have been highlighted through the study of both food deserts and food swamps, which examine a community's proximity to the physical location of healthy and unhealthy food outlets [24, 26–31]. Such studies have similarly found that the presence or lack thereof of healthy outlets may impact one's physical health and dietary intake [27, 32, 33]. Further, food access is often highlighted as a significant issue in more rural locations in the United States, where a community's nearest grocer may be 10 + miles away [34]. Studies examining such communities often emphasize both the physical limitations that living in such communities poses and the added benefits of new grocers when placed within a particular community [31, 34–37]. The current study underlines the importance of not only having healthy food outlets near families' homes but also considering how families get to and from these food outlets.

This study also adds to the understanding of food security and healthy food access as an intermediary social determinant of health [38]. From here, researchers may be able to examine transportation's relationship with food access within a more local context. There may also be policy implications for stakeholders on how to deliver interventions for those with low and very low food security while considering potential limitations this population may have in terms of transportation.

We found that only 3.8% of primary shoppers for families in the United States do not use a personal vehicle for grocery shopping—but 8.6% of family shoppers in low-income households rely on other means of transportation. Targeted interventions would benefit populations who are overrepresented in terms of food insecurity and lack of vehicle ownership. This may include increasing access to a vehicle through special programs that provide vehicles at a lower cost to those in need or providing alternative forms of transportation at the convenience of the population it hopes to benefit. Additional options include bringing healthful foods to the overrepresented populations through food delivery or creating healthy and affordable grocery options near their home.

### Strengths and limitations

This study has several strengths. First, results are generalizable to primary shoppers in households with children nationally. Stakeholders may be able to extrapolate information about this population to inform future research and policies. Additionally, food insecurity was measured using the standard USDA food security module, similar to previous research in this area [22, 39]. There were also several limitations to this study. As only about 4% of primary shoppers did not use a personal vehicle for these activities, sample sizes in this group were modest, especially within food security levels (sample Ns 12–18), and even more so when focusing on low-income families (sample Ns 10–16 within food security levels). Results should be interpreted considering this limitation. Additionally, this study relies upon cross-sectional data. Such data may be susceptible to response or recall bias. It also limits the ability to establish a causal or temporal relationship between the association of food insecurity and vehicle ownership.

In a nation as large and diverse as the United States, it may be helpful to understand the role that location plays in different contexts. While these areas are few and far between, some cities in the United States provide adequate public transportation and walkable infrastructure [40]. In many areas, it is nearly impossible to complete the most basic errands without the use of a vehicle [41]. Examination of each of these settings is worthwhile to understand what particular barriers are relevant to the populations living there. Finally, access to a personal vehicle is closely aligned with one's household income. The inclusion of income into the adjusted model illuminated this association's strength. In a secondary analysis, we found that food security did not differ by transportation for families below 130% of the federal poverty line. The relationship between race/ethnicity and income has also been studied extensively, where the income disparities between White and Black or Hispanic households remain steady [42]. As shown in Table 1, a higher percentage of family shoppers in the United States who did not use a personal vehicle were Black (38.1%) or Hispanic (39.7%). Thus, these households may be impacted greatest by a system that relies upon personal vehicle ownership to remain food secure. As future research is completed to examine this relationship in more local settings, they may analyze how food security may be exacerbated by issues related to race, ethnicity, poverty, and vehicle ownership.

## Conclusions

We found that primary shoppers for US households with children who did not use a personal vehicle for grocery shopping were more likely to report low and very low food security. Additional research is needed to understand if this association may be worsened based on one's location in a food desert or food swamp, level of social capital, or household income. The United States will likely remain a car-dependent nation for the foreseeable future. For many, access to a personal vehicle may be the determining factor in what helps them maintain food security. Further research is needed to understand this relationship within individual communities. Economic and social policies are necessary to ensure that all people can secure quality food for their homes.

## Data Availability

Data are available via the NHANES website at https://wwwn.cdc.gov/nchs/nhanes/Default.aspx.

## Code availability

Code may be made available upon request.
